# Maximizing the Impact of e-Therapy and Serious Gaming: Time for a Paradigm Shift

**DOI:** 10.3389/fpsyt.2016.00065

**Published:** 2016-04-18

**Authors:** Theresa M. Fleming, Derek de Beurs, Yasser Khazaal, Andrea Gaggioli, Giuseppe Riva, Cristina Botella, Rosa M. Baños, Filippo Aschieri, Lynda M. Bavin, Annet Kleiboer, Sally Merry, Ho Ming Lau, Heleen Riper

**Affiliations:** ^1^Department of Psychological Medicine, University of Auckland, Auckland, New Zealand; ^2^Department of Paediatrics: Child and Youth Health, University of Auckland, Auckland, New Zealand; ^3^Netherlands Institute for Health Services Research (NIVEL), Utrecht, Netherlands; ^4^Department of Psychiatry, University of Geneva, Geneva, Switzerland; ^5^Department of Psychology, Catholic University of Sacred Heart, Milan, Italy; ^6^Applied Technology for NeuroPsychology Laboratory, Istituto Auxologico Italiano, Milan, Italy; ^7^Department of Psicología Básica, Clínica y Psicobiología, Castellón, Spain; ^8^Department of Personalidad, Evaluación y Tratamiento Psicológicos, Valencia, Spain; ^9^CIBER Fisiopatología Obesidad y Nutrición (CIBERon), Instituto Salud Carlos III, Valencia, Spain; ^10^Department of Clinical Psychology, Faculty of Behaviour and Movement Science, Vrije Universiteit Amsterdam, Amsterdam, Netherlands; ^11^Department of Psychiatry, VU University Medical Center, Amsterdam, Netherlands

**Keywords:** computerized therapy, serious games, implementation, cCBT

## Abstract

Internet interventions for mental health, including serious games, online programs, and apps, hold promise for increasing access to evidence-based treatments and prevention. Many such interventions have been shown to be effective and acceptable in trials; however, uptake and adherence outside of trials is seldom reported, and where it is, adherence at least, generally appears to be underwhelming. In response, an international Collaboration On Maximizing the impact of E-Therapy and Serious Gaming (COMETS) was formed. In this perspectives’ paper, we call for a paradigm shift to increase the impact of internet interventions toward the ultimate goal of improved population mental health. We propose four pillars for change: (1) increased focus on user-centered approaches, including both user-centered design of programs and greater individualization within programs, with the latter perhaps utilizing increased modularization; (2) Increased emphasis on engagement utilizing processes such as gaming, gamification, telepresence, and persuasive technology; (3) Increased collaboration in program development, testing, and data sharing, across both sectors and regions, in order to achieve higher quality, more sustainable outcomes with greater reach; and (4) Rapid testing and implementation, including the measurement of reach, engagement, and effectiveness, and timely implementation. We suggest it is time for researchers, clinicians, developers, and end-users to collaborate on these aspects in order to maximize the impact of e-therapies and serious gaming.

## Introduction

The rationale for internet interventions for mental health is commonly centered on the following premises:

– Mental disorders, such as anxiety and depression, are common, disabling, and costly (1).
– Evidence-based interventions have been developed; however, the majority of people who would benefit do not receive any treatment (2).
– Largely, this “treatment gap” is due to structural or health system-related barriers (such as costs and lack of trained therapists), and social barriers, such as stigma (3).
– Internet therapies can offer scalable approaches whereby large numbers of people can receive treatment and/or prevention, potentially bypassing barriers related to cost, location, lack of trained professionals, and stigma (4).

Systematic reviews and meta-analyses of randomized controlled trials of internet interventions and computerized therapies delivered off-line (e.g., via CDRom) for anxiety and/or depression have reported good evidence of effectiveness (5–7), with adherence rates from 26 to 76% (8).

Despite the robust rationale and promising evidence, relatively few evidence-based interventions have been implemented or made publicly available. Among those that have been implemented in naturalistic or “real-world” settings (i.e., outside of traditional trials), limited data regarding implementation, uptake, and impact have been published. Available data suggest that significant numbers of people may be interested in online mental health support. For example, the publicly available self-help program, MoodGYM, attracted approximately 38,000 registrants over a 14-month period (9), and the mental health app, Happify, had been downloaded between 100,000 and 500,000 times on Google Play as at 16 December 2015. Moreover, a high proportion of people who complete the self-assessments in such interventions have substantial symptoms (10, 11). Despite this, naturalistic use of internet interventions for mental health appears to be associated with high attrition (non-adherence or drop out from the intervention); with notably higher rates of attrition in implemented programs than in randomized controlled trials, even when the same program is used. For example, in field studies of MoodGYM, only 3.9% of public registrants completed at least three of the five modules, in contrast to 53.8% of participants in a controlled trial of the same intervention (12). Less than 7% of public registrants continued past two modules in a newer version of the program (9), and similar results were found with adolescents (13). In another example, only 23% of users of a PTSD coach app used it during the first month after download, and the median time spent per session was less than one minute (14).

Increasing human support has been a core strategy for enhancing adherence to online interventions (8, 15, 16). This is promising with many trials finding higher rates of adherence to supported interventions than pure self-help interventions (17), although this is not always the case (18). Regardless of the comparison to pure self-help, attrition is still a challenge for supported internet interventions. For example, in a recent independent study by Gilbody and others (19), even with weekly telephone support, non-adherence was such that there was no treatment gain for patients accessing computerized therapies (MoodGYM and Beating the Blues) over those allocated to primary health care alone. Moreover, given that part of the rationale for computerized therapies is their scalability and low barriers to helpseeking, alternative approaches to increasing engagement should also be considered.

**Figure 1:**
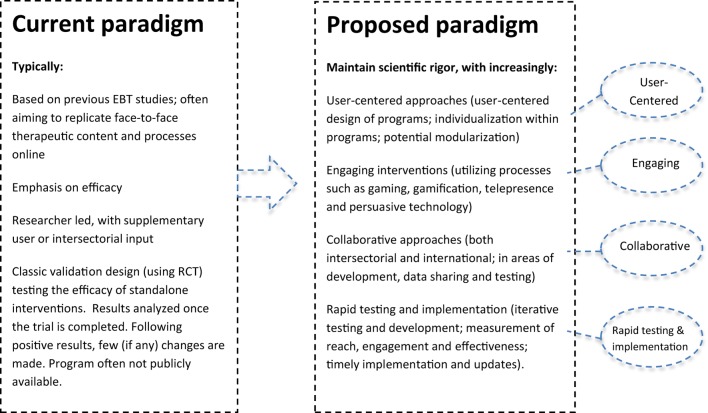
**Toward maximizing the impact of e-therapy and serious gaming for mental health**.

Together, findings from naturalistic or implementation studies suggest that potential users, including those with significant symptoms, are interested in internet interventions for mental health (20–23); however, implementation and engagement require improvement. We contend that the current paradigm or approach for the development of evidence-based internet interventions for mental health is typically researcher led; that the programs are often designed to replicate tested face-to-face therapies online; and that these are usually tested using classic validation designs (randomized controlled trials), with a focus on the efficacy of stand-alone interventions. Many of these components are critical to demonstrate that internet interventions can be effective under trial conditions as shown in Figure 1. However, to increase the impact of serious games and e-therapies, further developments are required.

An international group initiated by the last author (Heleen Riper) met in Amsterdam and Valencia with students and game designers. The group included authors of the present paper and others involved in e-health topics, including the fields of serious gaming (SPARX, Michael’s Game) and virtual reality (EMMA, BUTLER), apps for depression (MOODBUSTER) and substance use (Stop-cannabis), and online interventions for suicide prevention (PITSTOP suicide). An international Collaboration on Maximizing the impact of E-Therapy and Serious Gaming (COMETS) was established. We identified four pillars that need stronger emphasis for advancing research and increasing the impact of e-therapies and serious gaming. As shown in Figure 1, these are:

1. Increased focus on user-centered approaches (including user-centered design of interventions, and user responsiveness or individualization within interventions).
2. Greater emphasis on engagement (utilizing processes, such as gaming, telepresence and persuasive technology, and incorporating measures of engagement).
3. Increased collaboration across geographical regions, sectors, and interest groups.
4. Rapid testing and implementation.

The present paper outlines these potential approaches for increasing the real-world impact of internet interventions. New emphases or approaches should enhance, not replace, rigorous research-based approaches.

## Toward a New Paradigm

### Increased Focus on User-Centered Approaches

We propose that one of the key ways of increasing the impact of internet interventions is through increasing the focus on user-centered approaches. This would include user-centered design processes and greater individualization within programs, with the latter perhaps utilizing increased modularization.

#### User-Centered Design

While some computerized therapies and serious games have been designed with significant user input, we contend that uptake and adherence to internet interventions can be enhanced with greater involvement and understanding of users. This is not merely about consulting users on drafts but also about a deep understanding of user needs and preferences, and actively involving users in design processes from the outset. For example, a traditional research-centered process might begin with reviewing published evidence and subsequently planning an internet intervention containing six to ten modules to be completed at a rate of about one per week, hereby approximating evidence-based face-to-face therapies. By contrast, a user-centered design would begin with users, to understand issues such as how and when they would be willing to use the internet for mental wellbeing, and to explore their current behavior, needs, and preferences. Such an approach might suggest alternative processes or content. For example, alternative frequencies and durations of use might be proposed in order to reflect how people actually utilize the internet for psychological needs. Alternative processes for engagement or therapeutic change could also be identified for investigation (such as the use of sharable content or the opportunity to help others). The evidence for each of these components should, of course, be investigated. The point being not to replace research with user-centered design, but to utilize user-centered processes alongside scientific research.

#### Increasing Individualization

Alongside an increased focus on user-centered design *of* interventions, increased individualization, responsiveness, and choice *within* interventions may be important for engagement. To date, most evidence-based internet interventions for mental health are not very individualized. That is, all people using the same program generally all receive the same content, albeit sometimes with some optional modules. This is out of keeping with contemporary personalized experiences of the internet, which is very choice based. Engaging clients in defining their goals may result in better compliance than a more clinical and generalized aim of treating their diagnostic condition (24, 25). It should also be simple for users to select, from effective alternatives, their own preferred options or approaches for meeting goals. Modularization of interventions is promising in this regard.

#### Exploration of Modular Approaches

Limitations of current disorder-focused approaches to psychological therapies have been increasingly recognized (26). Briefly, most evidence-based psychological therapies have been developed for single disorders, such as depression, anxiety, and so on. However, in clinical practice, co-morbidity is the rule. In addition, there is an overlap in the techniques used to treat different disorders. In order to make treatments more efficient and to deal with clinical realities, a modular approach for face-to-face therapy has been developed, and early clinical trials show promise with better clinical outcomes delivered in less time (26–29). Should modularization prove effective, this approach would be feasible online, and could facilitate increased user choice and increased collaboration between groups.

### Increasing Engagement

A second key area for increasing the impact of internet interventions will involve the use of approaches that motivate continued usage (adherence) and improve user engagement (30, 31). The use of serious gaming and gamification, enhanced telepresence, and increased use of persuasive technology are promising in this regard. Moreover, the routine assessment of engagement may help to further develop the field and monitor progress toward this goal.

#### Serious Gaming and Gamification

“Serious games” are interventions that are games, or that utilize elements of gaming, as an integral and primary method for achieving a serious purpose, such as a health or educational goal (32). Gamification refers to the addition of gaming elements (such as challenges, reward, and experiences of exploration) to a non-game environment. The inclusion of gaming elements within computerized psychotherapies, and games or game-like environments with embedded therapeutic content, has been tested in several trials (32, 33). This approach is at an early stage, with few (if any) trials performed independent of developers (32, 34). However, there is promising evidence for serious games in other areas of health and behavior change (35–38). The potential for mental health has been identified (10, 39–44), and is supported by relatively low attrition rates in initial trials of mental health interventions utilizing these strategies (10, 45, 46).

#### Enhanced Telepresence

A therapeutic relationship is arguably a critical “active ingredient” of therapy (47). Increasing human support for users of internet interventions, via telephone, text, email, or face-to-face contact, appears to be helpful (8, 15, 16). Increasing social “telepresence,” or the feeling of connections with others within the computer program itself (48), may also hold promise. This can be achieved by using thoughtful design processes; for example, in the SPARX computerized CBT program, the “Guide” was designed as a virtual therapist, with warm welcoming wording, a carefully selected voice actor, and active rapport building. In interviews, young people reported feeling that the guide and other virtual characters in the computer program cared about them, and that this enhanced their experience of the intervention (49).

#### Increased Use of Persuasive Technology

The science of persuasive technology refers to the use of technology to influence human behavior, motivation, and attitudes through human–computer interaction or computer-mediated communication (50). Examples of persuasive technology include the use of automated support to increase primary task completion, such as automated SMS or mobile phone messages, email prompts, continued feedback by the program, and built-in explanations of why the program might help (51). Ethical issues related to the use of persuasive technologies must be carefully taken into account. Persuasion should be based on consent (52), and should help people to change the behavior they would like to change. Furthermore, not all participants require or prefer the same amount (or the same kind) of support, and this assistance may only be needed at critical times during the treatment program (53). Nevertheless, persuasive technology has been significant in promoting engagement and behavior change in other areas, and has arguably been underused in the development of internet interventions for mental health.

#### Measuring Engagement

A critical variable for improving the impact of computerized therapy interventions is patient or user engagement. According to Patient Health Engagement (PHE) models (54), making patients highly engaged in their care sustains them in attributing full meaning to the therapy, and in enacting self-management behaviors effectively, even when life contexts change. When effectively engaged, patients also develop a sustainable perspective about their actual and possible conditions, which can be better integrated into action (55). In this way, patient engagement can be considered as a compass to help developers customize their interventions. Patient engagement also has another advantage for developers and researchers: it can be easily measured using validated instruments (see Table 1).

**Table 1:** **Available validated tools for assessing Patient Engagement**.

Tool	Description
Patient Activation Measure (PAM) (56)	An interval-level, unidimensional Guttman-like measure with 22 (long version) or 13 (short version) items measuring self-assessed knowledge about chronic conditions, beliefs about illness and medical care, and self-efficacy for self-care. The PAM focused on physical conditions, and it was designed to measure activation as a broad construct
Health Confident Measure (HCM) (57)	A scale from 1 (low confidence) to 10 (high confidence). Used to determine a patient’s level of engagement and develop an individualized approach to managing care
Patient Health Engagement (PHE) Scale (54)	A 7-point, 5-item scale measuring patient engagement. According to the PHE model’s process view of patient engagement, individuals may be differentially engaged in one out of four levels of engagement – blackout, arousal, adhesion, and eudaimonic project – according to their emotional, cognitive, and behavioral mindset

### Rapid Testing and Implementation

#### Rapid Prototyping and Testing

Many health care interventions are developed after first consulting the literature, and a small number of experts and consumers. They are often then piloted within small groups, after which minor adjustments may be made before the intervention is tested in a larger trial. It can, therefore, be a number of years from initial development until the results of a controlled trial are published. Replication may be required, and only then does implementation become a priority. This drawn-out process is problematic when testing e-health interventions due to the speed of technology change. Alternative models of rapid development and iterative testing should be considered (58); for example, using agile software design principles, such as the lean start-up method (59) or scrum (60).

In agile development processes, the product is tested with users from the outset using rapid development and testing feedback loops. An important component involves the development of a minimal viable product (MVP). An MVP is a barely finished product that contains an essential element, but is missing details, and is provided to end-users to gage their reactions and inform the next steps in development. Reponses to the product are measured and used to inform next steps that are rapidly developed and tested in the same way. This iterative process involves close collaboration between designers, software developers, and end-users. Larger scale testing gradually replaces small opportunistic samples as progressively more complex features are tested. When a near-finished version is ready, more traditional testing can be carried out, for example, via a randomized controlled trial. As described by Mohr et al. (61), internet interventions can utilize approaches that focus on evaluating the working mechanisms, rather than a locked-down version of the intervention. Such a framework allows for improvements in functionality to be made during a trial, subsequently resulting in a more generalizable and durable intervention.

#### A Planned Focus on Implementation

Many internet interventions for mental health have been developed and shown positive results in trials, but are not publicly available, or, are available, but have limited uptake or adherence. This highlights two points. First, that efficacy alone is not sufficient to indicate an intervention will have a significant impact on health. Reach (or exposure to and uptake of the intervention) and adherence must also be evaluated, and findings used to improve programs and their implementation (62). Second, the necessary conditions for the sustainable implementation of interventions, including consideration of the project’s future ownership and the identification of possible revenue streams for ongoing hosting and updates, must be considered from the outset. Implementation may, therefore, necessitate non-traditional collaborations.

### Increased Collaboration

#### Intersectorial

A key opportunity for improving the impact of computerized therapies and serious gaming is increased use of diverse knowledge and skills. The design of internet interventions for mental health should involve input from different fields, including – but not limited to – users, therapists, computer engineers, game designers, behavior change experts, and human factors specialists. Such collaboration requires researchers to move beyond their discipline and consider new knowledge, methods, and techniques. Current approaches for evidence-based internet interventions for mental health are often initiated and led by researchers. Alternative approaches, for example, where researchers join projects that are led by users, game developers, or internet and software experts, should also be considered.

#### International Collaborations

International collaboration is a further key opportunity for improving the long-term sustainability and growth of e-therapy. Many internet interventions are developed within a specific country or jurisdiction, and fail to make use of the international nature of the internet, with subsequent limitations in terms of funding and the impact of the proposed intervention. These stand-alone programs are often supported by modest financial resources, resulting in small-scale clinical trials or case studies. A promising strategy is to develop stronger multinational teams and projects.

#### An Ambitious Future Vision

Should increased collaboration and modular user-centered approaches be pursued, greater gains in population mental health may be realized. This could be achieved, for example, by creating a user-centered online platform or ecosystem that allows users to select the components that most appeal to them or that are recommended based on their self-assessments. Should such a model become a reality, components could be continuously developed and additional ones added over time. A well-designed system could allow uptake, adherence and effectiveness of components to be routinely measured and compared. We propose that, in the future, such systems could safely invite user-generated content and input from other researchers and developers within agreed guidelines. Should this vision be achieved, the input and energy of diverse groups could be harnessed to facilitate development in the field.

## Conclusion

Evidence-based mental health interventions are promising; however, uptake and adherence outside of trial settings have not yet met hopes and projections. We have proposed that it is time for a paradigm shift in order to maximize the impact of evidence-based e-therapies and serious gaming. Promising directions include a greater focus on user-centered approaches (including user-centered design, individualization within interventions, and exploration of modularized programs), increased emphasis on engagement (utilizing processes such as gaming, telepresence and persuasive technology, and measuring engagement), increased international and intersectorial collaboration, and rapid testing and implementation. We propose that, in the future, such systems could safely invite both user-generated content and input from other researchers and developers. In each case, input should be within agreed guidelines.

## Author Contributions

HR initiated the COMETS group. CB, RB, GR, AG, YK, TF, DB, AK, HL, and SM developed or contributed to the concepts of the COMETS group, which are represented in this paper. TF, DB, YK, AG, GR, CB, RB, FA, and LB drafted the paper. AK, SM, HL, and HR contributed substantial content to the paper. All co-authors approved the paper.

## Conflict of Interest Statement

TF and SM are co-developers of SPARX computerized therapy for depression and can benefit from any commercialization of it outside of New Zealand. The remaining authors have no conflict of interest to declare.
